# An attitude network analysis of post-national citizenship identities

**DOI:** 10.1371/journal.pone.0208241

**Published:** 2018-12-03

**Authors:** Raphaela Schlicht-Schmälzle, Volha Chykina, Ralf Schmälzle

**Affiliations:** 1 College of Education, Michigan State University, East Lansing, MI, United States of America; 2 The Donia Human Rights Center, University of Michigan, Ann Arbor, MI, United States of America; 3 Department of Communication, Michigan State University, East Lansing, MI, United States of America; Institut Català de Paleoecologia Humana i Evolució Social (IPHES), SPAIN

## Abstract

How are evaluative reactions pertaining post-national citizenship identities interrelated and what are the potential mechanisms how post-national identities evolve? Previous efforts to operationalize and measure post-national citizenship identities leave it open how people’s stances on different issues are related and suffer from a variety of theoretical and methodological shortcomings regarding the nature of political attitudes and ideologies. A recently proposed approach conceptualizes ideologies as networks of causally connected evaluative reactions to individual issues. Individual evaluative reactions form the nodes in a network model, and these nodes can influence each other via linked edges, thereby giving rise to a dynamic thoughts system of networked political and identity-related views. To examine this system at large, we apply network analysis to data from the European Values Study. Specifically, we investigate 33 evaluative reactions regarding national and supra-national identity, diversity, global empathy, global environmentalism, immigration, and supra-national politics. The results reveal a strongly connected network of citizenship identity-related attitudes. A community analysis reveals larger clusters of strongly related evaluative reactions, which are connected via bridges and hub nodes. Centrality analysis identifies evaluative reactions that are strategically positioned in the network, and network simulations indicate that persuasion attempts targeted at such nodes have greater potential to influence the larger citizenship identity than changes of more peripheral attitude nodes. We lastly show that socio-demographic characteristics are not only associated with the overall level of post-national citizenship, but also with the network structure, suggesting that these structural differences can affect the network function as people develop national or post-national citizenship identities, or respond to external events. These results provide new insights into the structure of post-national identities and the mechanism how post-national identities might evolve. We end with a discussion of future opportunities to study networked attitudes in the context of civic and citizenship education.

## Introduction

People simultaneously belong to local, regional, national, and supranational political entities (e.g. a citizen of Barcelona, Catalonia, Spain, the European Union, the world), and they internalize these social relationships in the form of citizenship identities. Over the last few centuries, the nation has become the base level to which citizenship identities become predominantly attached [1,2]. Critically, the recent trend towards globalization with its economic, political, and sociological effects has promoted citizenship identities that exceed national borders, including incorporating ‘global citizenship education’ into the United Nations Sustainable Development Goals [3], often to the dismay of nationally focused politicians and citizens [4]. The European Union is another example of a strong attempt to elevate not only politics and trade, but also citizenship identities beyond nationality [5–8]. Recent events, such as Brexit and the success of national protectionist parties in Europe and elsewhere, describe a backlash against globalization and a recurrence of national identities, coupled with resistance against an integration of citizenship identity beyond the nation [9,10]. People seem to be divided by the scope of their citizenship identities [11,12]; recent societal conflicts, such as the refugee crisis or various economic disputes, make it salient how much it matters how individuals think and feel about their citizenship attachment [13–15].

The main goal of this article is to understand how such post-national citizenship identities are structured and through which mechanisms they evolve. We therefore explore how evaluative reactions to a broad set of political and identity-related statements are interrelated in a network and how they may mutually interact in a networked manner.

We first review the literature on post-national citizenship identities and related concepts and discuss the measurement of political attitudes and ideology. We then introduce and apply network analysis to data from over 30,000 respondents of the European Values Study, which includes many questions relevant to national and different aspects of post-national citizenship identities, which prompt evaluative responses from people based on the views they hold on the issues in question.

Broadly speaking, post-national citizenship refers to a subjective sense of oneself as a member of humanity at large as opposed to a social groups defined by nationality [16–19]. Official passports codify the legal membership in a nation state, but people may vary in how much value they assign to it and how central it becomes to their identity [20]. That evaluative reactions pertaining to citizenship are inherently subjective is particularly evident with regard to post-national citizenship identities: Symbolic passports of *the world*, although existing, do not have far-reaching legal implications. In fact, none of the variants of codified post-national citizenship guarantees rights and obligations [16,17,21] even remotely comparable to those that come with national citizenship.

Nevertheless, some people regard themselves as citizens of the world more than others do. Borrowing ideas from social psychology [20,22–24], citizenship can be seen as the subjective conception that one belongs to, identifies with, and the feelings and cognitions towards respective members of in- and outgroups one has [20,21].

As of today, most individuals feel first and foremost as citizens of a nation. This is barely surprising since it is the nation state that provides all its citizens with equal rights and obligations [1,2,25,26,27]. However, these national identities are neither a natural kind nor cast in stone. Rather, over the past centuries, newly arising nations used their strong political powers to orchestrate a ‘mass educational enterprise’ [1] to form and promote strong national identities [27–29]. Perhaps not unlike during the early years of the nations, globalization challenges existing borders and identities [26,30]. In sum, national citizenship ‘has become a thing’, but post-national citizenship identities remain in a nascent state, and how individuals construct their citizenship identities in the future will have powerful effects on their political preferences and on global politics [31–33].

Previous efforts to address post-national identities have in common that they refer to an ideology that is somewhat incompatible with an exclusive national identity. Scholars however distinguish between various forms of post-national citizenship—world, global, cosmopolitan, transnational, supranational, and post-national citizenship identities, or European citizenship among others [16,17,34–37]—each focusing on different aspects or levels of post-national identities and some even potentially conflicting. Especially with regard to European citizenship it is questionable whether it is an initial steps to overcome nationalism on the way towards ‘global citizenship’ or whether a further integration of European citizenship only replaces nationality by a ‘fortress of Europe’ identity that is strongly demarcated by European borders and therefore rather a constraint of ‘global citizenship’ identities [21,38]. Such questions however, can only be solved by empirical studies acknowledging the complexity of citizenship identities and that explore how individual evaluative reactions (for example empathy with Europeans, empathy with people of the own nation, and humanity at large) are interrelated. To explore the complex relations between a wide variety of evaluative reactions we apply the anchor term ‘post-national identities’ as it covers any form of citizenship identity that is not exclusively defined by the nation [17]. The goal of this paper is to analyze how a broad variety of attitudinal aspects that are often associated with post-national identities are interrelated and can thus dynamically influence each other.

Typical aspects related to ‘post-national’ identities include a ‘sense of belonging to all humanity’ or ‘to a social group beyond the nation’, ‘confidence in supranational politics and institutions’ such as in the United Nations or the European Union [2,4,17,19,39], or an ‘awareness of global interdependence and interconnectedness’ for example with regard to the global environment or the economy [40]. Further, ‘a broad scope of empathy with human fate around the world’ [2], and a ‘willingness to accept ethnic, cultural, and racial diversity’ in the own social context [21,41,42] are seen as core elements of a post-national citizenship identity. These descriptions sound plausible, but lack specificity as they are not attitudes toward concrete objects but involve further abstract concepts. The same applies to negative definition attempts, which state that post-national citizenship is not compatible with nationalist and cultural protectionist views and outgroup derogation based on national, ethnic, religious, or cultural background [43–45]. The role of patriotism and a general strong affinity to social group identities (e.g. to religious communities or to membership in organizations) and whether it automatically leads to outgroup derogation remains controversial [46–49]. Overall, literature lacks a clear empirical statement of how different evaluative reactions contribute to a post-national identity and how they are interrelated. Our empirical results contribute to the understanding of the mechanisms how post-national citizenship identities evolve and which attitudinal aspects are main forces for the their development.

## Attitude networks

Most previous work on different forms of post-national citizenship identities leaves the central question still unanswered: How are evaluative reactions pertaining post-national citizenship identities interrelated and how do individuals build these identities? Answering this question requires first zooming in on the concept of attitude and its two political allies, beliefs and ideologies. Historically, the tripartite model of attitudes has suggested that attitudes are latent variables (e.g. attitudes towards immigrants) that are expressed through specific evaluative reactions (e.g. ‘immigrants are straining the welfare system’ or ‘immigrants undermine the national culture’) towards an attitude object, such as people from different social groups [50]. These evaluative reactions can be cognitive, affective, and behavioral [51–54]. Much research has examined how attitudes are structured within a so-called attitude system, or ideology, and how they are related to ensure attitude consistency and ideological coherence [55–58]. In doing so, most research has relied on reflective measurement models, which posit latent constructs that are often subsumed under single nouns like liberalism, conservatism, authoritarianism, environmentalism, or citizenship identity [59]. This suggests that behind the measured responses lies a hidden thing (‘-ism’) that *causes* to maintain certain attitudes and ultimately single evaluative reactions [60,61]. In the case of ‘post-national citizenship’, for instance, individuals would approve of open borders, or empathize with immigrants *because* of their magnitude of ‘post-national citizenship’. All causation derives from the latent entity and changes in single attitudes and evaluative reaction only occur because of a change in the overall latent ideology (citizenship identity).

The assumptions of conceptualizing attitudes and ideologies as latent variables have long been challenged as unrealistic [59,62] and criticism has been mounting across the social sciences more broadly for years. This is, for example, expressed in the quote of Feldman & Johnston [59] that “[…] ideology cannot be reduced to a single value or measure which accurately represents the political beliefs of all citizens. We believe that empirical examinations of liberalism and conservatism that disregard such complexity in meaning and structure fail to detect some important aspects of the determinants of ideology and their ultimate consequences for politics (p. 338).” Over the years, several authors have suggested that ideologies represent networks in which attitudes are linked to each other and form a belief system [55,59,62–67]. According to this conceptualization, a change in one evaluative reaction can cause change in other related evaluative reactions as well [68–70], as individuals strive for attitudinal consistency [71,72]. This conceptualization can have far reaching implications for persuasion attempts and the prediction of change of ideological states [62]. While the notion of belief systems is very compatible with the notion of networks, research on attitudes and political ideology has rarely utilized network analyses.

Over the past few years, network theory emerged as an interdisciplinary framework to understand a wide range of phenomena that proved difficult to reconcile with latent variable models, such as mental disorders, personality, intelligence, and lately also attitudes [73–76]. Applying network theory to attitudes, the Causal Attitude Network (CAN) model [76] conceptualizes attitudes and ideologies not as entities based on a common cause, but rather as networks in which measurable evaluative reactions represent nodes that are causally connected through edges [77–79]. The construct of an ideology is defined through its attitudinal elements and their systematic connections [80]. In this framework, ideological states such as post-national citizenship arise through the interactions between evaluative reactions that together strengthen or weaken the picture of post-national citizenship. Individual nodes (evaluative reactions) can exert forces along network’s connections (edges). For example, if one thinks that immigrants undermine the national culture, this could make one also think more easily that there are too many immigrants and that they take jobs away, which consequently leads to a downwards spiral in post-national citizenship attitudes. This reasoning is compatible with the notion of striving for cognitive consistency, a prominent concept in research on attitudes and persuasion [81,82].

In the remainder of this article we explore the network structure of attitudes associated with post-national citizenship. We apply the term ‘post-national citizenship’ as an umbrella term for different varieties of post-national citizenship such as global citizenship or European citizenship and take a rather broad perspective on attitudes commonly associated with citizenship identities that overcome nationality [16,17]. The resulting network provides a snapshot of how individuals’ evaluative responses to European Values Study statements relate to each other. We examine the network structure through key network diagnostics, simulate how citizenship attitude networks might reconfigure as a result of political events or persuasion attempts, and test whether socio-demographic variables are associated with both the level of post-national citizenship and the structure of citizenship attitude networks.

## Data and methods

The data for this project come from the European Values Study [83], a large cross-national cross-sectional survey of the values, attitudes, and dispositions that European citizens hold. This makes this dataset well-suited for our purpose of examining post-national citizenship based on the new network perspective on attitudes. We focus on post-national citizenship identities in Europe since its attempts to establish a post-national identity through the European Union create a unique testbed to examine citizenship identities. Our sample includes data of 39,030 respondents in 27 European OECD member states. The focus on OECD member states within Europe ensures relative homogeneity with regard to economic development. The countries included are: Austria, Belgium, Czech Republic, Denmark, Estonia, Finland, France, Germany, Great Britain, Greece, Hungary, Iceland, Ireland, Italy, Latvia, Luxembourg, Netherlands, Northern Ireland, Norway, Poland, Portugal, Slovak Republic, Slovenia, Spain, Sweden, Switzerland, and Turkey.

After a wide literature search on post-national citizenship, we selected 33 items that are relevant to citizenship identities and are asked in the 2008–2010 wave of the European Values Study (S1 Appendix). The questions address participants confidence in national and supra-national institutions, patriotic feelings and social-group identity, the kinds of communities (far and close distance) participants feel empathetic with, the stances towards immigrants and immigration, their approval of diversity with regard to several minority groups, or the sense of responsibility for the global environment. Descriptive statistics show that most of the evaluative reactions maintain a well-balanced distribution in the sample (S1 Table). Each of the 33 evaluative reactions will define a node that is then statistically related to the other nodes, and the strength of this relationship is mapped to an edge between the two nodes. Thus, with 33 nodes, there are 528 possible edges. To aid the interpretation of results, we re-coded all nodes so that value 1 indicates predisposition towards post-national citizenship, whereas 0 denotes absence or opposition against post-national citizenship. For example, value 1 on the node D1 ‘empathy with Europeans’ would mean ‘presence of empathy with Europeans’ while 0 means ‘not much empathy with Europeans’. Importantly, the coding direction does not have any impact on the structure of the network but rather on the direction of the edges and is thus structurally equivalent. The original variables are binary, or 4 to 10 point scales as displayed in columns 4 and 5 of S1 Appendix.

Following the Causal Attitude Network (CAN) model of Dalege [76], we apply the eLasso procedure based on the Ising [84] model [85–87] (S2 Appendix). The eLasso procedure performs l1-regularized logistic regression models for all 33 attitude variables in which each node is iteratively regressed on all other 32 nodes as independent variables [86]. The edge weights in the estimated network are the logistic regression parameters between two nodes, controlled for all other nodes [85,86]. To demonstrate the robustness of this network we conduct bootstrap analyses [88]. We also analyze different functions of the nodes in the network structure, namely how groups of nodes form clusters or communities or related attitudes, and whether some nodes are more centrally located than others and have a greater potential to influence the overall belief system.

## Results

### General network structure

**Fig 1:**
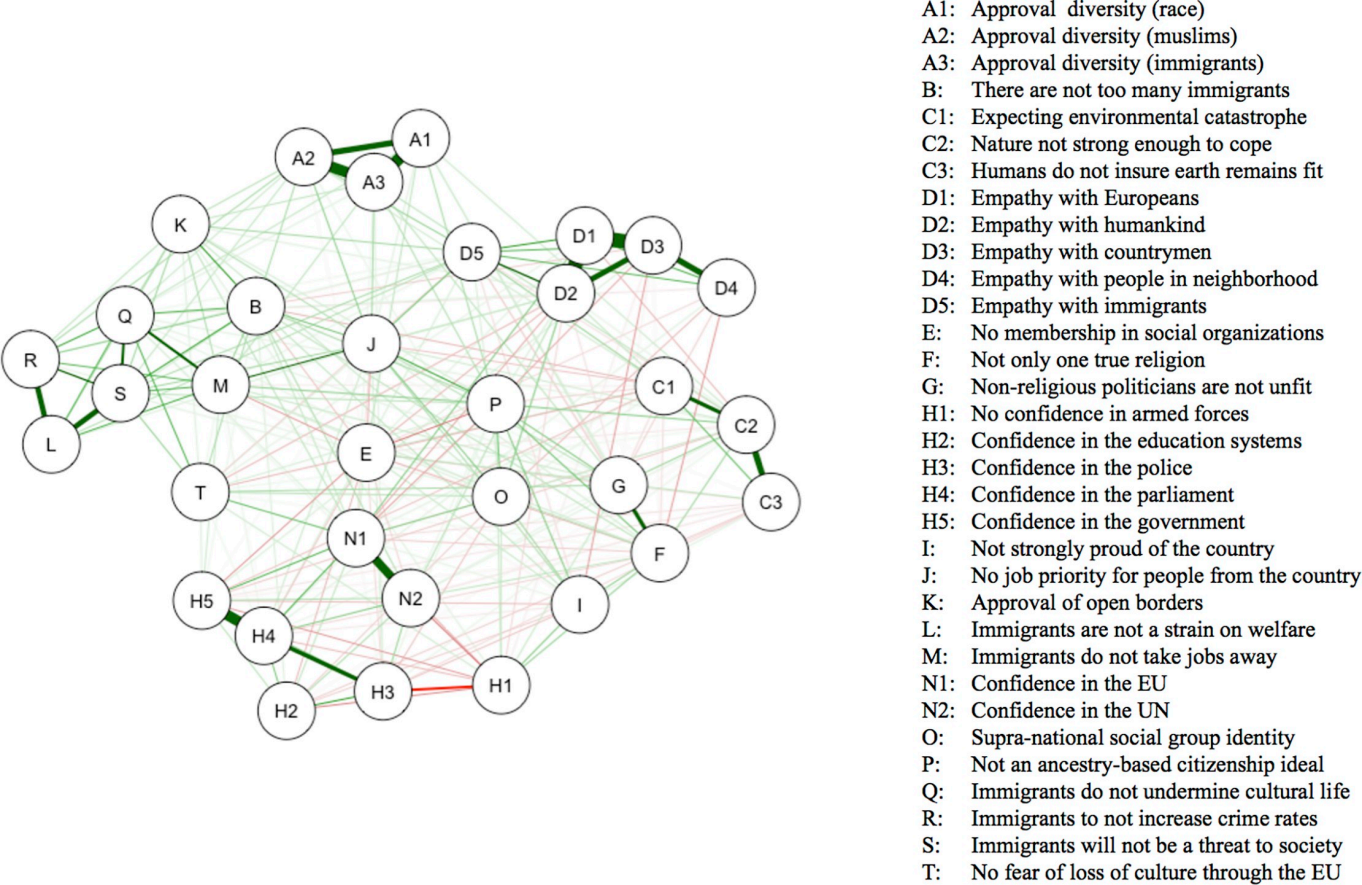
Network structure of post-national citizenship attitudes. The nodes display the individual attitudes, and the edges represent relationships between nodes. Green edges represent positive relationships, red edges represent negative relationships, and the thickness of the edges corresponds to the strength of the relationship.

The network analysis reveals a strongly connected network related to post-national citizenship, which is shown in Fig 1. All 33 nodes are connected to the network, suggesting an integrated system with nodes as elements. This system together comprises ‘post-national citizenship’, and the nodes—no matter whether they rather relate to ‘European citizenship’ (e.g. empathy with Europeans or confidence in the European Union) or to ‘global citizenship’—are mainly positively related to each other. To confirm the stability of the network, we computed a nonparametric bootstrap analyses using the R bootnet function for edges and node centrality across 1000 iterations (S1 and S2 Figs) [88]. The bootnet function tests whether the network edges remain stable across multiple (in our case 1000) sub-samples of the original sample. The corresponding plot (S1 Fig) reveals the bootstrapped confidence intervals around each of the estimated edge weights and shows whether the edges are stably positive or negative.

**Fig 2:**
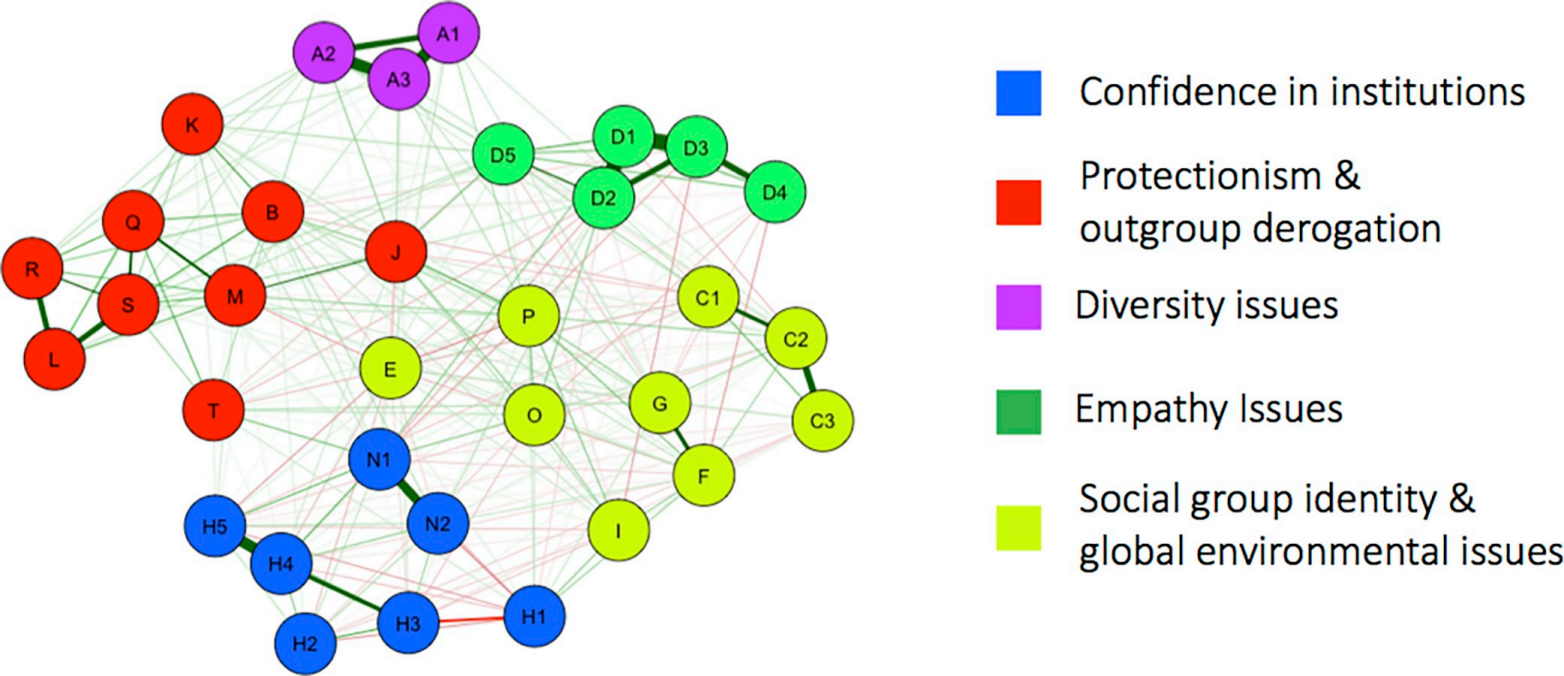
The network community structure. A community analysis reveals 5 clusters of nodes that are more related to each other.

Networks often contain several clusters, which are examined through community analysis [89]. That is, some nodes have rather dense connections with each other and relatively sparser connections to other nodes, akin to groups and cliques in social networks. A community analysis of the network with the walktrap community finding algorithm [85] identifies five distinct clusters of attitude items, which are represented as different colors in Fig 2. Feedback loops among these evaluative reactions are thus especially likely. We tentatively label the first cluster (Cluster 1, blue) as ‘confidence in institutions’. The emergence of this cluster shows that confidence in different kinds of political and administrative institutions—no matter whether national or supranational—are positively related. The second cluster (Cluster 2, red) includes nodes that describe ‘protectionist and outgroup derogative views’, including one of the most central nodes J (job priority for natives). Cluster 3, plotted in lime green, contains nodes associated with a weak or strong ‘(national) social group identity’, but also includes the nodes describing ‘awareness of global environmental problems’ (C1-C3). Nodes that describe ‘local and global empathy’ comprise a fourth cluster (Cluster 4, green), underscoring the relatedness of both local and global empathy. The final cluster (Cluster 5, purple) describes the ‘approval of diversity’ in one’s social context. Although these communities are somewhat separable, they are also connected by bridges (e.g. between nodes J and P in Fig 2) implying a strong global connectivity of the network. A small world structure in attitude networks means that the networks show a combination of strong clustering with strong global connectivity, such that clusters are connected through bridges, which facilitates short routing of information [76,90,91]. We computed a small-world index of 1.27 for our unweighted network based on Dalege et al. [65] and Humphries et al. [92]. To test whether the small-world index is higher than 1, what is indictive for a small-world structure, we computed the confidence intervals of random graphs using 1,000 Monte-Carlo simulations. The upper limit of the 99.9% confidence interval for the corresponding random graphs was 1.07. The clustering of the citizenship identity network is 0.65 while the clustering of the corresponding random network is 0.51. The Average Shortest Path Length of our citizenship identity network is with 1.49 equal to that of the corresponding random graph.

Dalege et al. [76,93] argue that a highly connected attitude network indicates high attitude strengths. Of note, in highly connected networks, the attitudes are more stable and difficult to change, which requires more persuasive force since the strongly related evaluative reactions keep each other in place.

### Centrality analysis and network dynamics

**Fig 3:**
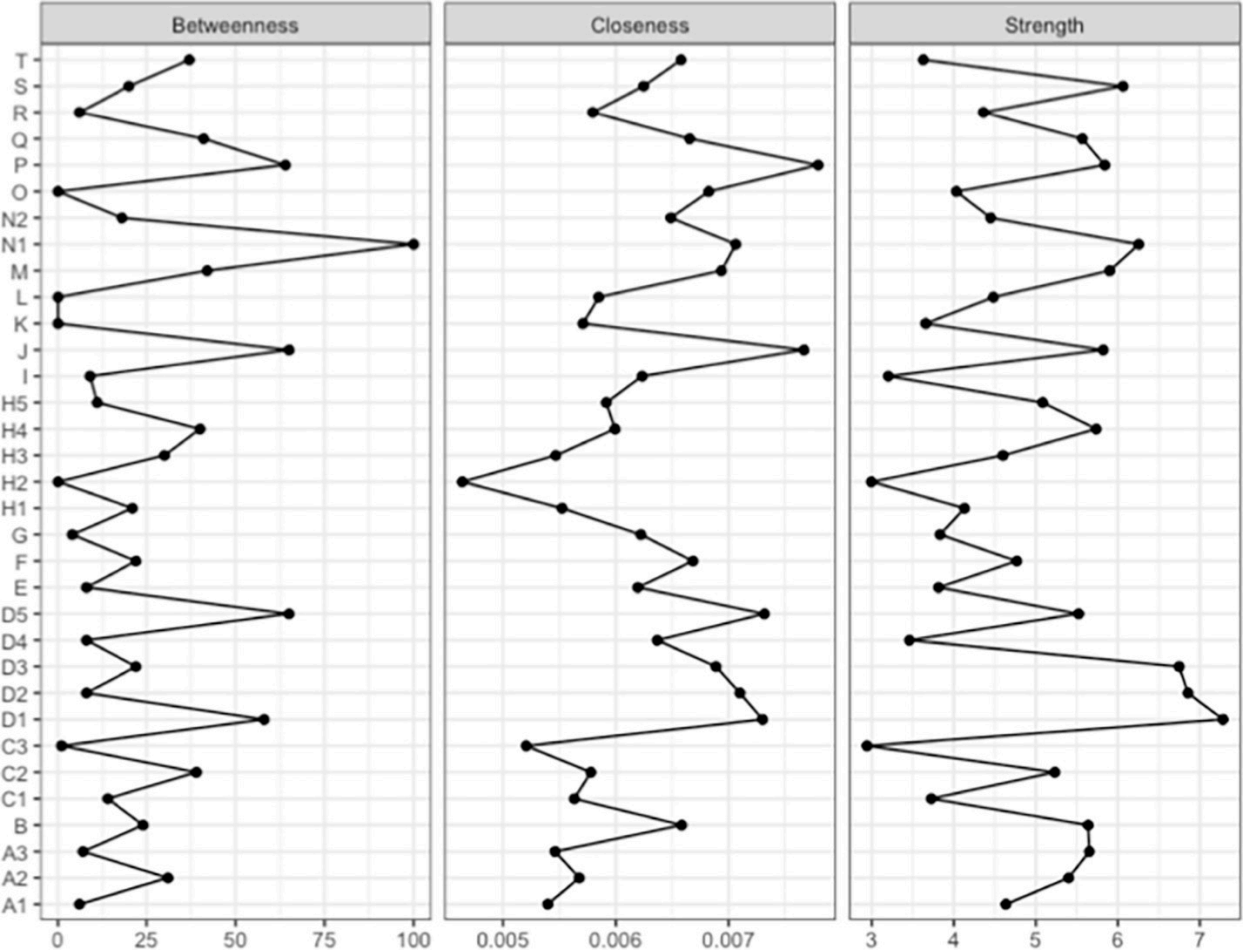
Node centrality. Centrality indices for Betweenness, Closeness, and Strength centrality for all 33 nodes. For details see text.

Next, we examined which nodes occupy central positions in the network and may thus be better positioned to spread information across the whole network (Fig 3). The most common centrality measures are strength, closeness, and betweenness [85,94–96]. Strength is the sum of the edge weights that are directly connected to a given node and describes the direct impact a given node has on the network. Closeness relies on the Average Shortest Path Length as it is the inverse sum of the shortest paths a specific node is connected to each of the other nodes in the network. Especially closeness therefore describes the implications of a given node for the spread of information through the whole network. Betweenness, also grounds on the Average Shortest Path Length but describes the number of average shortest paths a given node is placed and thereby can interrupt the flow of information throughout the network. Based on closeness centrality, the most central nodes are P (ancestry-based citizenship ideal) and J (job priority for natives). The node ‘confidence in the European Union’ (N1) maintains a strong betweenness and therefore can disrupt the spread of information in the network. Thus, rather nationalist and protectionist views may spread less easily through the network if a person has strong confidence in the European Union. As for the edge-stability analysis, bootstrap analysis confirms that centrality indices are stable across subsets of cases in the sample (S2 Fig). To examine the stability of centrality measures, the bootnet function reduces the sample to a fraction of itself in an iterative manner. Even for a sample of 30% of the original sample, the sample centrality measures are strongly related to the entire sample’s centrality (*r* > 0.5). According to Epskamp et al. [88], this stability is displayed by the maximum percent of cases that can be dropped of the sample without resulting in a correlation of the original centrality scores of lower than 0.7 (with 97% certainty). S2 Fig. shows that these stability criteria are met for all three stability measures even for a sample of 30% of cases of the original sample.

**Fig 4:**
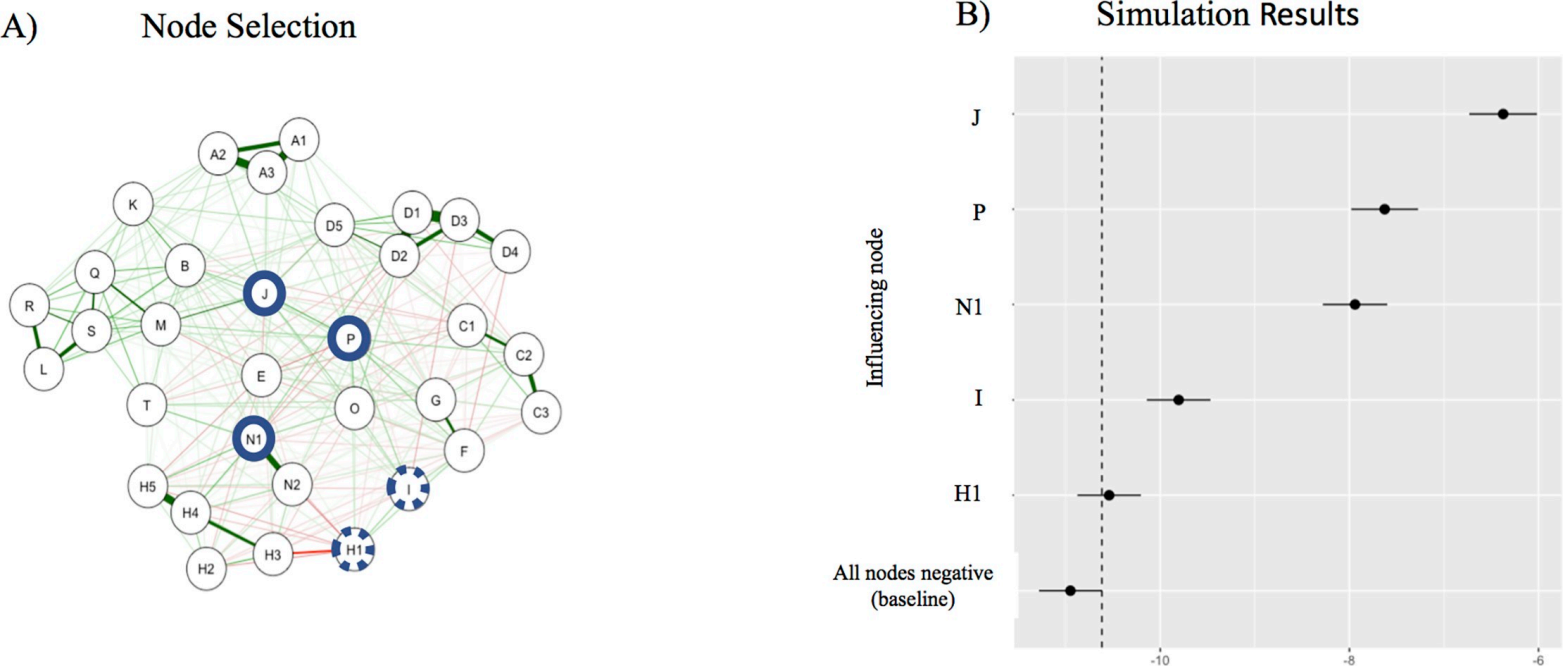
Sum scores of the simulation samples. The effect of changes in single nodes towards a positive disposition on the overall network sum scores (including 95% confidence intervals) compared to a simulation sample in which all nodes have a negative disposition (jittered line).

A core feature of the network approach is that complex attitudinal states like ideologies arise from interactions between evaluative reactions within a network [75]. Given that our data is cross-sectional, we cannot determine with certainty whether the centrally located attitudes have a causal impact on temporal network dynamics. However, using the IsingSampler function in R we can simulate persuasion attempts on more centrally and less centrally located nodes in our network and form model-based predictions of how this would spread across and change the network [85] (S3 Appendix). Let us assume we focus on individuals with moderately negative attitudes towards post-national citizenship. We can simulate such a group of individuals with moderately negative evaluative reactions on all our network nodes of post-national citizenship by setting the thresholds of all nodes (likelihood of a node to take the value 1) to a moderately negative value (-0.1). The mean sums core of this network is accordingly very low (negsample in Fig 4). The sum score indicates the overall level of post-national citizenship, computed by summing over all items. We now want people to increase their overall sum scores in the post-national citizenship network by targeting our persuasion attempts (let’s say a civic education class) at node J (‘job priority for natives), seeking to convince them to refuse ‘job priority for natives’. We simulate this persuasion attempt by a sample in which node J has a strongly positive thresholds (1.0) while the thresholds of all other nodes remain moderately negative (-0.1). In this sample, individuals have a strong likelihood to refuse job priority for natives. The sample reveals a significantly higher sum scores than the sample with all nodes having a negative threshold (SampleJ in Fig 4). To show how nodes differ in their consequences for the whole network depending on their centrality, we repeat our simulation by targeting more centrally and less centrally located nodes. In each simulation we increase the threshold of a single node (e.g. J) to a positive value (1.0) and leave the thresholds of all other nodes at a negative disposition. Fig 4 shows that the impact the persuasion attempt on a single attitude node has on the overall network differs between more centrally and less centrally located nodes. The simulations, in line with network theory, preliminary indicate that centrally located nodes (J, P, and N1) have a stronger impact on the overall network than peripherally located nodes (H1 and I). In sum, a persuasion attempt on attitudes such as ‘an ancestry-based citizenship ideal’ or ‘job priority for natives’ should have a stronger impact on the overall post-national citizenship identity than an attempt targeting ‘confidence in the armed forces’ or ‘strongly proud of the country’.

### Comparing networks of citizenship attitudes based on socio-demographic characteristics

**Fig 5:**
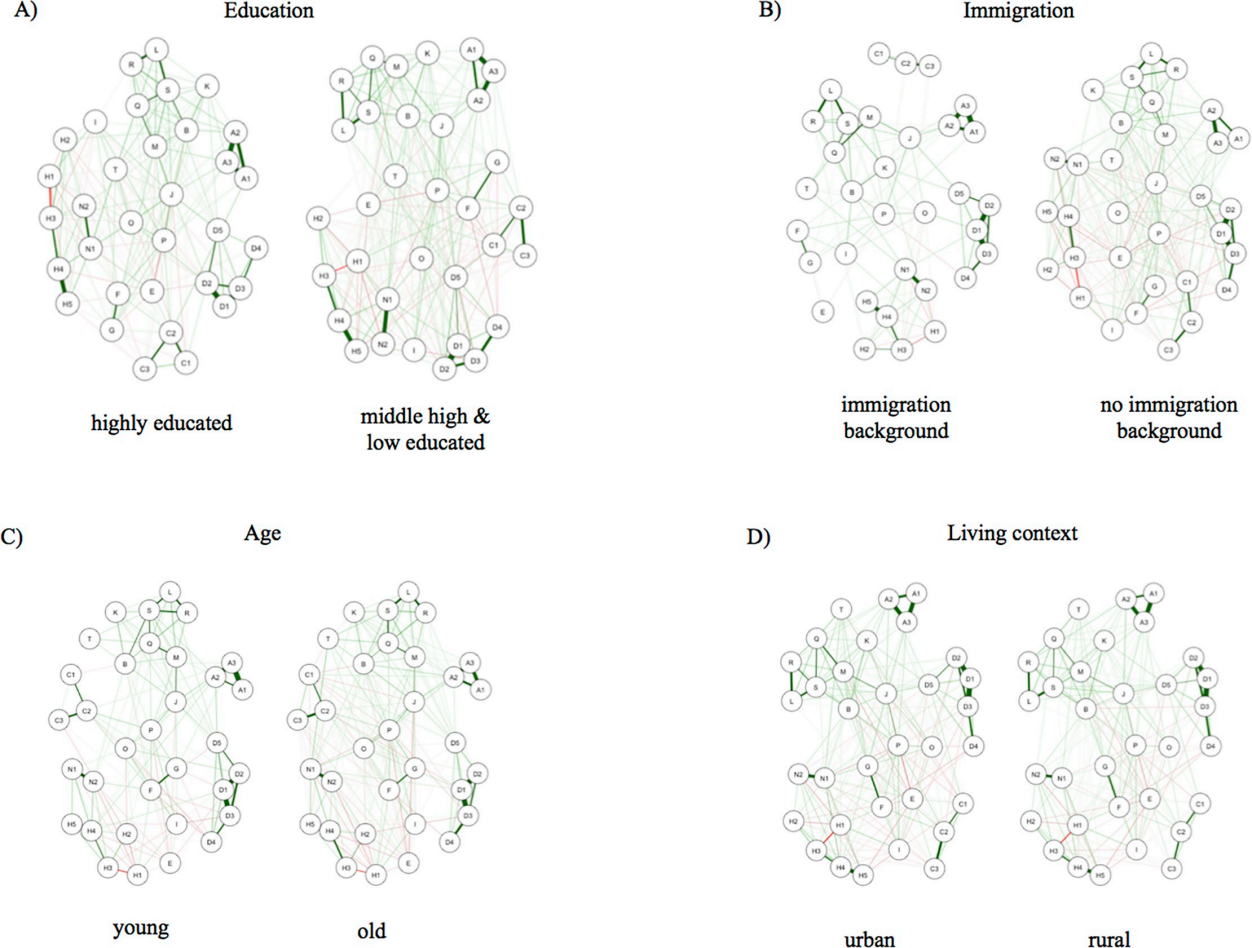
Comparison of citizenship attitude networks by socio-demographic characteristics. Network structures by socio-demographic background: for education (A), immigration background (B), age (C), and urban vs rural living context (D).

Many attitudes co-vary with socio-demographic factors. Previous studies have shown that the state of post-national citizenship varies between people from different socio-demographic backgrounds, with younger [97,98] and more educated individuals [98,99], as well as those living in urban environments [98], being more likely to obtain post-national citizenship identities. Further, mobility and migration increases individuals’ propensity to post-national citizenship identities [100–102]. By comparing the mean sum scores of our network by socio-demographic factors, we can confirm these findings (S3 Fig). Beyond these relatively broad comparisons, however, attitude network analyses allow us to examine whether network structures differ for these socio-demographic groups, such as whether the post-national citizenship attitudes of younger and older people connect differently within their belief systems, thus potentially indicating different pathways by which post-national citizenship identities arise and change. Fig 5 displays citizenship networks computed for individuals from different socio-demographic backgrounds. A Network Comparison Tests (NCT function in R) [87,103] formally tests whether the network structure differs significantly. We therefore run a Network Comparison Test for a) individuals with an immigrant background vs. individuals without an immigrant background, b) highly versus low to moderately educated people, c) urban living vs. rural living individuals, and d) old versus younger people. The Network Comparison Test cannot deal with missing values. We therefore, compute two different tests for each socio-demographic category: a first test that drops all cases that obtain missing values and a second one where we imputed all missing values based on complete cases with the ‘logreg’ method of the mice function in R. Both tests show similar results. The tests reveal significant network structure differences based on the level of education and familial immigration background (S4 Appendix). For example, in the network of low to moderately educated people, nodes F ‘there is only one true religion’ and H1 ‘confidence in the armed forces’ are connected, but not for highly educated people. In the network of individuals with an immigration background, nodes A1-A3 (approval of diversity) are barely connected to the network at all, while they are strongly connected in the network for individuals without an immigration background. On the other hand, NCT does not detect differences between the attitude networks of urban vs rural people and older versus younger people in our data.

## Discussion

The main goal of this article is to explore how attitudes relevant for post-national citizenship are interrelated in a network and how they mutually interact. Globalization changes how individuals conceive of national borders and citizenship identities. Recent events like Brexit or the increasing relevance of national protectionist parties—not only within Europe—make it salient that individuals vary in the geographic scope of their citizenship identities and how this influences their political decisions and lastly leads to impactful governmental transformations. Somewhat ironically, the nation states were once themselves invented to overcome scattered regionalism and its problems [1,104].

Post-national citizenship, most specifically global citizenship and global citizenship education, is often touted as a potential remedy against social problems arising as side effects of globalization. According to social identity theory [105–107], post-national citizenship identity should protect against feelings of threat to one’s national in-group identity that result from migration and mobility [48,108,109]. This hypothesized beneficial social function pertaining to conflict prevention and resolution is what motivates efforts to promote ‘global citizenship education’ around the world. Similar arguments have been laid out regarding economic and environmental benefits of positive views on ‘post-national citizenship’ issues: That citizens with post-national identities are aware of their global interconnectedness and interdependence and therefore may sense a responsibility for the global environment and act to reduce global poverty and inequality [110]. To better understand and potentially forecast the forces that influence macro-societal attitudes, it is vital to examine how individuals’ citizenship identities are structured, how information may spread throughout attitude systems, and how external events could impact these networks.

Our findings suggest that attitudes commonly associated with post-national citizenship identity are closely interrelated and form a cohesive ideological belief system. This first of all shows that the often-claimed elements of post-national citizenship are not just an arbitrary set of attitudes but a system of a related evaluative reactions that can interact with each other and can produce feedback loops [76,85]. This means that becoming a citizen beyond the nation is not as simple as accruing a certain set of attitudes–a post-national citizenship identity is more likely to arise when certain combinations of attitudes either occur or do not occur within an individual. As such, the formation of a post-national citizenship identity is not an additive but rather an interactive process. The strong interconnectedness of the attitudes associated with post-national citizenship also indicates high attitude strength and an ideological belief system that is rather stable and can only change under strong persuasive force [76,93].

Beyond studying the citizenship network as a whole, we identify five groups of closely related attitudes that cluster in communities. These attitudes coalesce around issues related to global and local empathy, confidence in national vs. supranational institutions, appreciation of diversity in the own social context, attitudes towards national protectionism with regard to immigration, and sensing a belonging to a national in-group and interdependence in the global environment. Within these clusters, the nodes are very strongly connected and a spread of information within these clusters is particularly likely. The network is further characterized by strong clustering and strong global connectivity as clusters are connected by bridges. This shows how different attitudinal domains within post-national citizenship (but potentially also beyond) are connected and how ideological comorbidity arises. A noteworthy substantive difference to the latent variable model is that the network model does not conceptualize these clusters as independent (orthogonal) dimensions. Interactions between variables that load on different factors are theoretically difficult to reconcile with latent factor models. Moreover, variables that would load strongly on two clusters (such as our nodes J and P) would be seen as weak indicators of a latent factor, which stands in stark contrast to the network model in which these nodes are located in central positions that enable them to orchestrate information flow throughout the post-national citizenship attitude system.

Two nodes that are noteworthy due to their central positions are the ‘ancestry-based citizenship ideal’ and ‘desiring job priority for natives’. Both nodes are overall strongly connected to the rest of the network. A strong ‘confidence in the European Union’ is positioned on many shortest paths in the network. Thus, if a person has strong confidence in the EU, it is hard to spread information that decreases post-national citizenship in all its forms throughout the network, while for individuals who do not have ‘confidence in the EU’, it may be difficult to adopt opinions that lean towards post-national citizenship. The results also indicate that attitudes associated with different gradations of post-national citizenship are positively related, meaning that at this point of time in history, individuals who have strong confidence in the European Union also are more likely to share attitudes that are more generally related to ‘global citizenship’ attitudes While several scholars expect strong European identities as a constraint for ‘global citizenship’ these results indicate the opposite at least for the current state. Other nodes, such as ‘confidence in the armed forces’ or ‘general patriotic feelings’ are more peripheral.

Given that network structure underpins and constrains network function, changes in centrally located nodes can induce changes within the broader network [75]. Network dynamics arise when either individual experiences or external events, such as societal conflicts, media reports, or education, trigger a change in one (or more) attitude node(s). For example, a media report about the fate of refugees might evoke empathetic responses and thus prompt a dynamic reorganization of related attitude structures in one way, whereas watching a documentary about decaying local industries may set off a cascade in the opposite direction. Such spreading activations [111,112] within an individuals’ attitude network are most likely in strongly connected attitude networks [65]. Simulations of persuasion attempts targeted on more centrally and less centrally located nodes predict that targeting centrally located nodes—especially with a high closeness centrality (‘ancestry citizenship ideal’ and ‘desiring job priority for natives’)—is most effective in increasing or decreasing the overall picture of post-national citizenship throughout the network. In particular the centrality of the node ‘desiring job priority for natives’ speaks for an ongoing importance of economic competitiveness as a stymie for citizenship identities that cross national borders. Both central nodes describe cases of moderate in-group favoritism [113–115]. This challenges recent result of social psychology that speak for harmless forms of national in-group dynamics that are unrelated to strong forms of national outgroup discrimination [49].

Socio-demographic background indicators do not only influence the overall picture of post-national citizenship, but also the network structure and thus the mechanisms by which citizenship identities operate. By comparing the sum scores of the network between individuals of different levels of education, immigration experiences, ages, and urban or rural living conditions, we find support for common hypotheses about their impact on post-national citizenship identities [97,110]. More educated people have higher sum scores than medium or less educated people, younger people have higher scores than older people, urban living individuals have higher scores than rural living individuals, and individuals with an immigration background in the family have higher scores than their peers without immigration experience. However, above and beyond these global differences, the results show that also the network structures—the way how attitudes are connected within the network—varies systematically with the education level and the immigration experience: People with high education levels or an immigration experience in the close family seem to connect attitudes—or strive for attitude consistency—differently than people without these characteristics. Thus, socio-demographic determinants such as education or immigration experience may not only affect the overall level of post-national citizenship, but also the internal structure of ideologies and belief systems and how individuals connect different attitudes.

The network approach lays out a viable path to empirically analyze complex and interrelated attitudes and ideologies. The system-related thinking that is inherent to the network approach holds great potential to better understand the complex relationships between individual issues and particularly the dynamics that unfold between them as they change and influence each other, giving rise to emergent dynamics within an individual but also on a societal level that are “more than the sum of parts”.

Our work could barely touch on these issues, which are only superficially addressed by the simulation analysis. We chose the European Values Study because of the breadth of attitudinal questions that the survey has, as well as a large cross-national sample of the study. Our data, however, also has limitations. More specifically, the cross-sectional data do not allow us to identify whether the relationships between the nodes that we observe are of a causal nature. The causal nature of the relationships between the nodes is however a central element of the Network Theory, especially when it comes to predicting the network dynamics. Moving forward, it will be critical to follow up on this by conducting longitudinal **(**temporal) analyses of network changes. Such analyses may either be carried out at a macro level with information from media content analysis, or with experimental designs on the individual level using techniques such as ecological momentary assessment [116]. Recent advances in computational social sciences offer a wealth of methodological tools, which could be combined with the current network analyses to provide deep insights into pervasive issues in research on political attitudes and opinion dynamics [117,118]. Future work should explore these possibilities.

## Summary and conclusion

It has long been suggested that ideological identities have a network-like structure [55,63], but to our knowledge this article is among the first to formally examine this proposition. The study of identity formation and citizenship education could benefit from adopting network approaches to i) examine how individuals build their ideological states and how they change dynamically as well as ii) how external events intervene into individual belief systems but also affect macro-societal political trends.

## Supporting information

S1 Table Variables and binary coding. The node labels, the corresponding original variables from the European Values Study codebook, the binary coding strategy, as well as the descriptive statistics of the nodes.
(PDF)

S1 Fig Stability of Edge Weights. Bootstrap analysis of edge weights and respective 95% confidence intervals (horizontal axis) for all possible edges (vertical axis). The horizontal lines represent one of the 528 edges of the network. The labels on the vertical axis have been removed for the purpose of legibility. The red line are the sample estimates and the gray area the bootstrapped confidence intervals. Each horizontal grey line is an edge of the network.
(TIFF)

S2 Fig Centrality Stability. Bootstrap analysis of node centrality indices. Average correlations (vertical axis) between centrality indices of reduced samples and the original sample. Lines are the mean correlations and the colored fields indicate the range from the 2.5^th^ and the 97.5^th^ quantile of the correlations.
(TIFF)

S3 Fig Global Citizenship Identities by socio-demographic backgrounds. Network sum scores by socio-demographic background and their 95% confidence intervals.
(TIFF)

S1 Appendix R-code data preparation. This file contains the R code for our initial preparation of the European Values Study data for the further analyses as well as the link to the original third- party data provider. The code is also accessible on github.com/Raphaela82/GlobalCitizenship_EVS.
(RMD)

S2 Appendix R-code network analysis. This file contains the R code for the network analysis, network diagnostics, and robustness tests. The code is also accessible on github.com/Raphaela82/GlobalCitizenship_EVS.
(RMD)

S3 Appendix R-code network simulations. This file contains the R code for the simulations of network dynamics and persuasion attempts on nodes of different centrality. The code is also accessible on github.com/Raphaela82/GlobalCitizenship_EVS.
(RMD)

S4 Appendix R-code network comparison test. This file contains the R code for the network comparison test to identify differences in the network structure of global citizenship attitudes based on socio-demographic backgrounds. The code is also accessible on github.com/Raphaela82/GlobalCitizenship_EVS.
(RMD)
