# Supplementary material for: An attitude network analysis of post-national citizenship identities
Source: PLoS One. 2018 Dec 3;13(12):e0208241. doi: 10.1371/journal.pone.0208241 (PMC6277102; doi:10.1371/journal.pone.0208241)
Supplement: S1 Table — The node labels, the corresponding original variables from the European Values Study codebook, the binary coding strategy, as well as the descriptive statistics of the nodes. (PDF) [file pone.0208241.s001.pdf]

Node	Variable in EVS 2008 Codebook	Variable description based on codebook	Value 1 (global citizenship)	Value 0 (not global citizenship)	n value 1 (global citzienship)	n value 0 (not global citizenship)	% of 1 (global citzienship)	% of 0 (not global citzienship	NA in %
A1	v47	don't like as neighbours: people of different race	not mentioned	mentioned	32653	4879	83.66	12.50	3.84
A2	v53	don't like as neighbours: muslims	not mentioned	mentioned	30351	7074	77.76	18.12	4.11
A3	v54	don't like as neighbours: immigrants/foreign workers	not mentioned	mentioned	31221	6249	79.99	16.01	4.00
B	v275	immigrants living in your country: there are too many	disagree stromgly, disagree, neither agree nor disagree	agree strongly & agree	19459	17780	49.86	45.55	4.59
C1	v301	environment: if things continue we will experience a catastrophe will experience a catastrophe	agree strongly & agree	disagree strongly & disagree	28606	6329	73.29	16.22	10.49
C2	v299	environment: nature is strong enough to cope with impacts of industry	disagree strongly & disagree	agree strongly & agree	23902	10316	61.24	26.43	12.33
C3	v298	environment: human ingenuity insures earth remaining fit	disagree strongly & disagree	agree strongly & agree	13094	20498	33.55	52.52	13.93
D1	v288	are you concerned with livings conditions of europeans	very much & much	to a certain extent & not so much & not at all	5982	32237	15.33	82.60	2.08
D2	v289	are you concerned with livings conditions of all humans around the world	very much & much	to a certain extent & not so much & not at all	10214	28042	26.17	71.85	1.98
D3	v287	are you concerned with livings conditions of fellow countrymen	very much & much	to a certain extent & not so much & not at all	11062	27440	28.34	70.30	1.35
D4	v285	are you concerned with livings conditions of people in your neighborhood	very much & much	to a certain extent & not so much & not at all	14226	24472	36.45	62.70	0.85
D5	v292	are you concerned with livings conditions of immigrants	very much & much	to a certain extent & not so much & not at all	8631	29701	22.11	76.10	1.79
E	v25	do you belong to: none social organization	mentioned	not mentioned	16499	18343	42.27	47.00	10.73
F	v128	only one true religion or no religion offers any truths	there is not one true religion, but all great world religions contain some basic truths & none of the great religions have any truths to offer	there is only one true religion & there is only one true religion, but other religions do contain some basic truths as well	21459	14676	54.98	37.60	7.42
G	v134	politicians who do not believe in god are unfiot for office	disagree strongly & disagree	agree strongly & agree & neither agree nor disagree	24475	12833	62.71	32.88	4.41
H1	v206	how much confidence in: armed forces	not very much & not at all	a great deal & quite a lot	14123	23221	36.18	59.50	4.32
H2	v207	how much confidence in: education system	a great deal & quite a lot	not very much & not at all	26206	11569	67.14	29.64	3.22
H3	v210	how much confidence in: the police	a great deal & quite a lot	not very much & not at all	26818	11550	68.71	29.59	1.70

H4	v211	how much confidence in: parliament	a great deal & quite a lot	not very much & not at all	15466	21748	39.63	55.72	4.65
H5	v222	how much confidence in: government	a great deal & quite a lot	not very much & not at all	13557	23847	34.73	61.10	4.17
I	v256	how proud are you to be a ... (country) citizen	quite proud & not very proud & not at all proud	very proud	18299	17034	46.88	43.64	9.47
J	v102	when jobs are scarce: employers giving people from the country priority with jobs	disagree & neither	agree	14909	23204	38.20	59.45	2.35
K	v266	should people from less developed countries be allowed to come to the country for work?	let anyone come who wants & let people come as long as thee are jobs available	put strict limits on the number of foreigners who can come here & prohibit people coming here from other countries	18416	19120	47.18	48.99	3.83
L	v271	immigrants are a strain on welfare system	values 6-10 (rather siagree to disagree)	values 1-5 (rather agree to agree)	10651	26556	27.29	68.04	4.67
M	v268	immigrants take away jobs	values 6-10 (rather siagree to disagree)	values 1-5 (rather agree to agree)	15795	22251	40.47	57.01	2.52
N1	v214	how much confidence in: EU	a great deal & quite a lot	not very much & none at all	17381	18405	44.53	47.16	8.31
N2	v216	how much confidence in: UN	a great deal & quite a lot	not very much & none at all	18974	15392	48.61	39.44	11.95
O	Combination of v253 and v254	to which geographic group do you beloing first (v253) and second (v254)	either Europe or the world as a whole on at least one of the two variables (v253 and v254)	neither Europe nor the world as a whole on at least one of the two variable v253 or v254	8828	29270	22.62	74.99	2.39
P	v278	for citizenship it is important: to have [country nationality] ancestry	not important & not important at all	very important & quite important	15823	22486	40.54	57.61	1.85
Q	v269	immigrants undermine countrys cultural life	values 6-10 (rather siagree to disagree)	values 1-5 (rather agree to agree)	19106	18638	48.95	47.75	3.29
R	v270	immigrants increase crime problems	values 6-10 (rather siagree to disagree)	values 1-5 (rather agree to agree)	10324	27469	26.45	70.38	3.17
S	v272	immigrants will become a threat to society	values 6-10 (rather siagree to disagree)	values 1-5 (rather agree to agree)	12814	24216	32.83	62.04	5.12
T	v258	EU fears: lose national identity/culture	values 6-10 (not afraid at all)	1-5 (very much afraid)	15087	21408	38.65	54.85	6.50

n= 39030

Source: https://dbk.gesis.org/dbksearch/file.asp?file=ZA4800_cdb.pdf
