# Supplementary figures and images for: An attitude network analysis of post-national citizenship identities
Source: PLoS One. 2018 Dec 3;13(12):e0208241. doi: 10.1371/journal.pone.0208241 (PMC6277102; doi:10.1371/journal.pone.0208241)

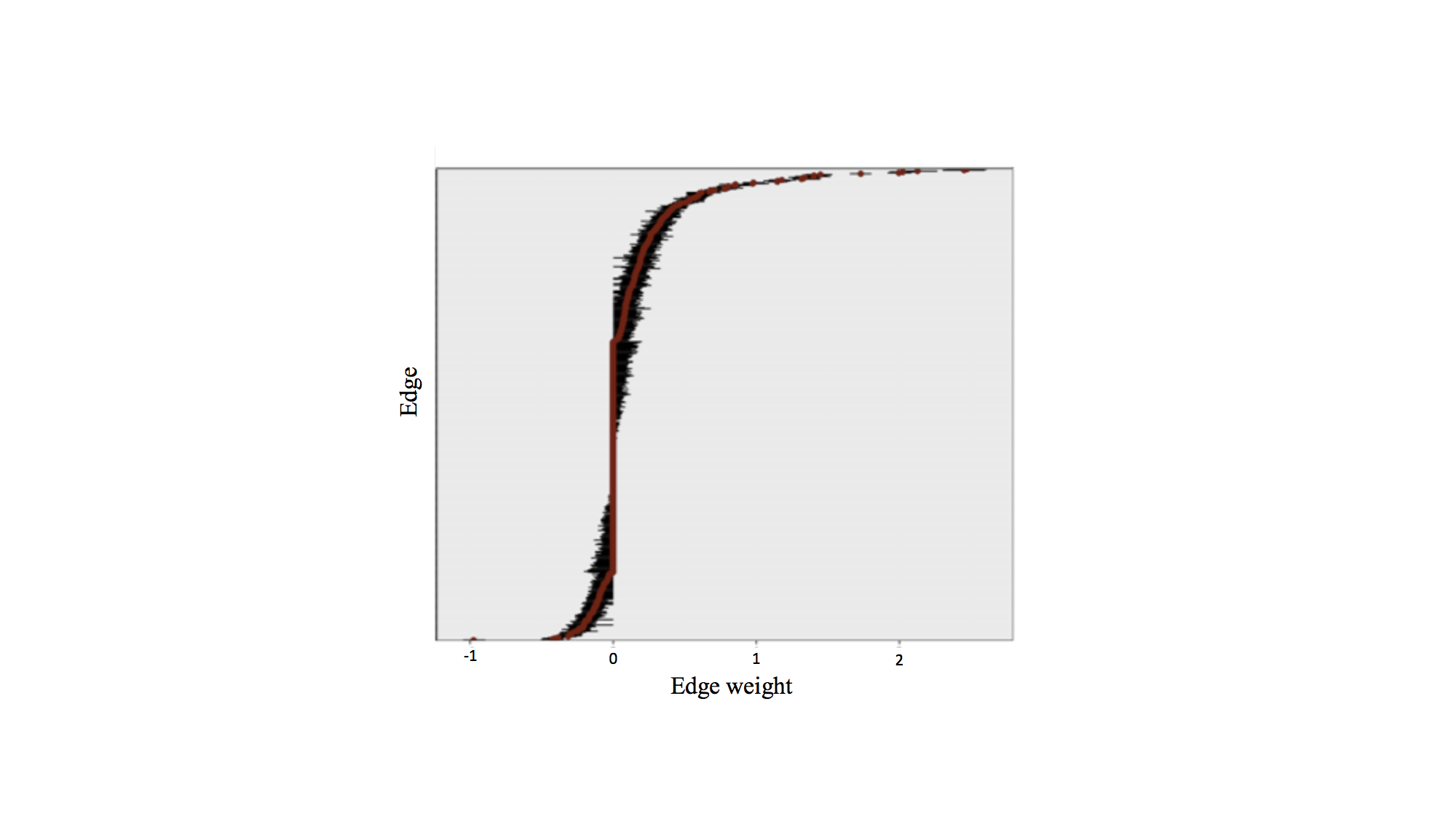

Supplement: S1 Fig — Bootstrap analysis of edge weights and respective 95% confidence intervals (horizontal axis) for all possible edges (vertical axis). The horizontal lines represent one of the 528 edges of the network. The labels on the vertical axis have been removed for the purpose of legibility. The red line are the sample estimates and the gray area the bootstrapped confidence intervals. Each horizontal grey line is an edge of the network. (TIFF) [file pone.0208241.s002.tiff]

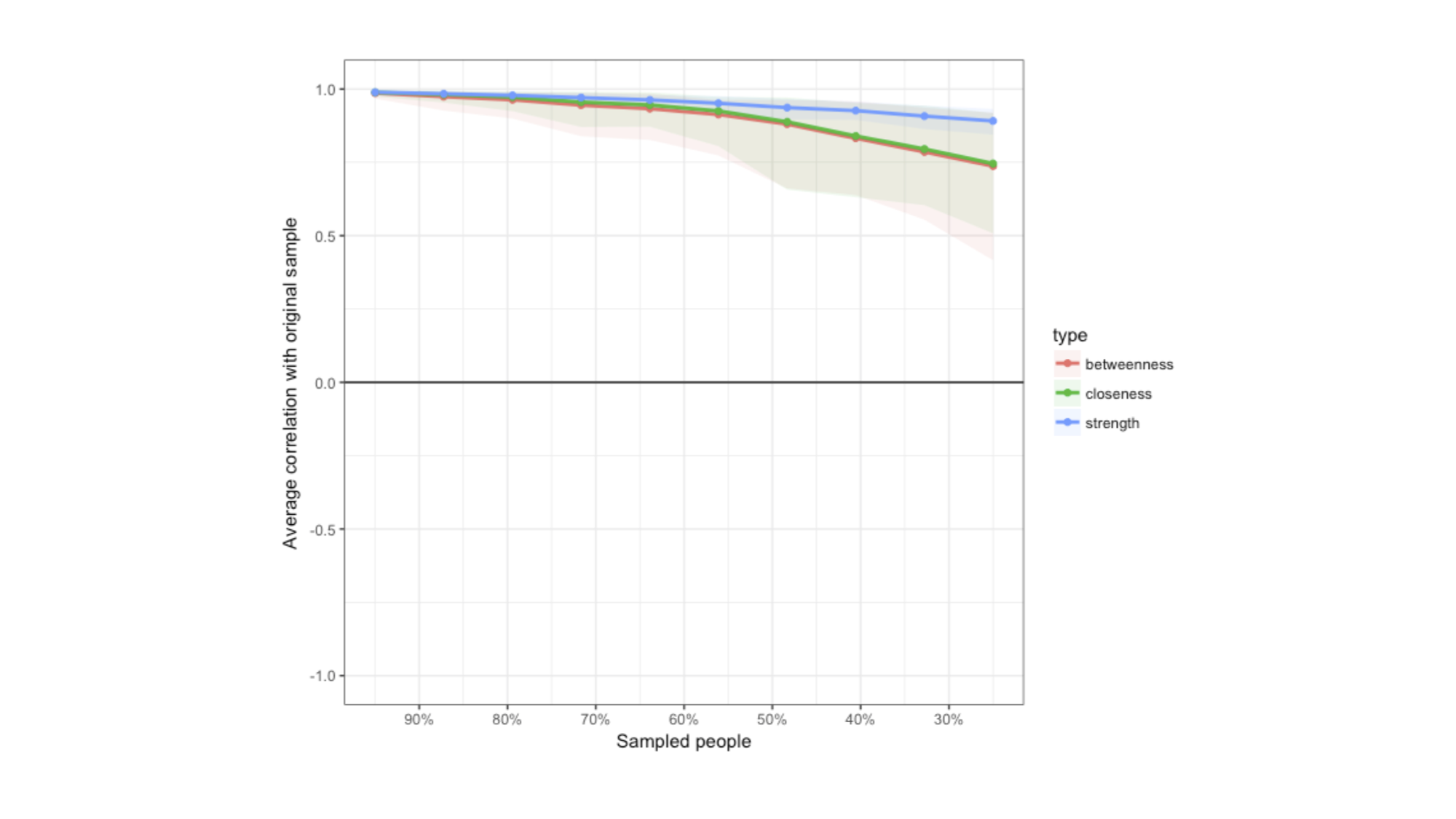

Supplement: S2 Fig — Bootstrap analysis of node centrality indices. Average correlations (vertical axis) between centrality indices of reduced samples and the original sample. Lines are the mean correlations and the colored fields indicate the range from the 2.5th and the 97.5th quantile of the correlations. (TIFF) [file pone.0208241.s003.tiff]

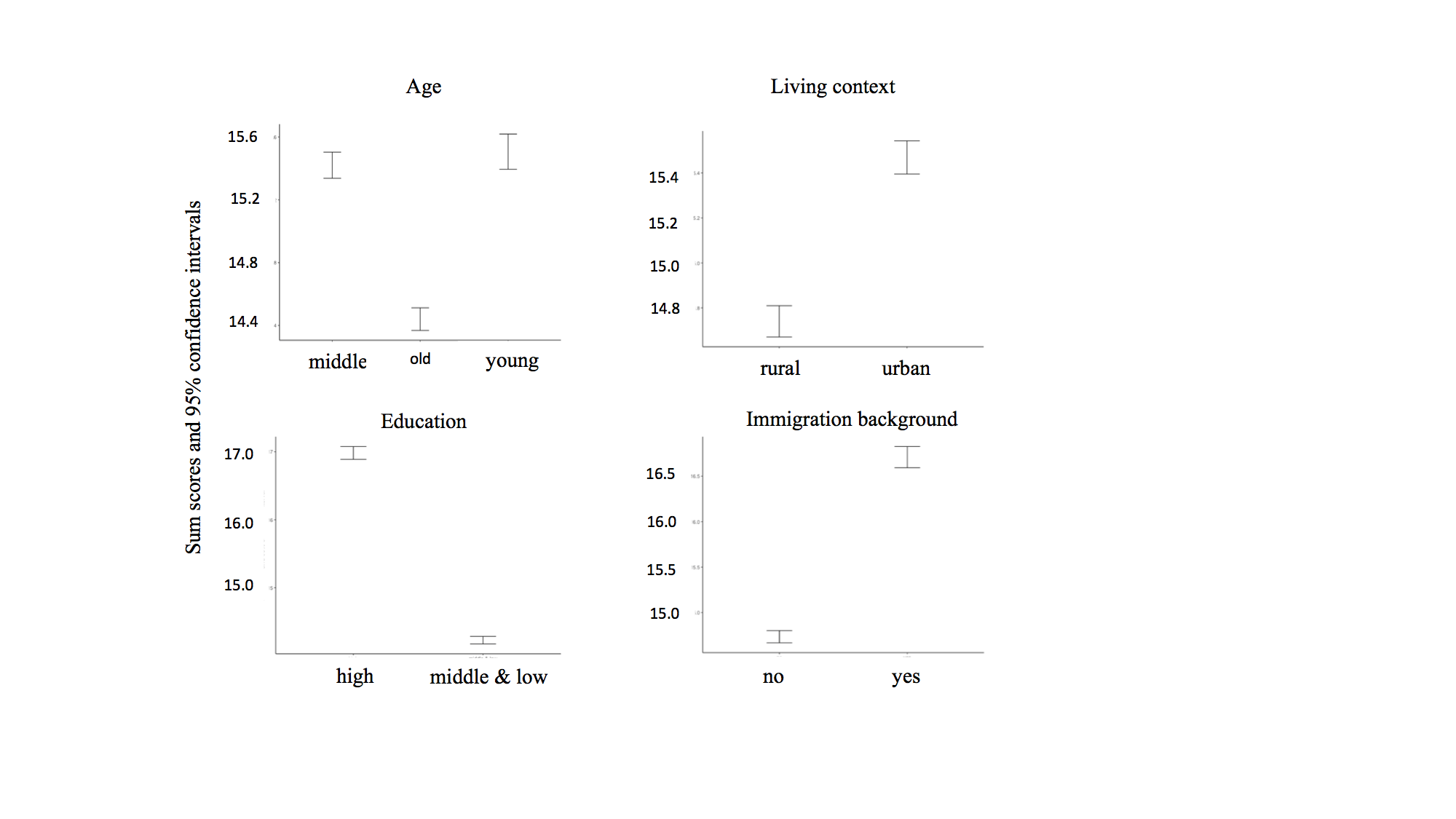

Supplement: S3 Fig — Network sum scores by socio-demographic background and their 95% confidence intervals. (TIFF) [file pone.0208241.s004.tiff]
